# Acyl Ghrelin Induces Insulin Resistance Independently of GH, Cortisol, and Free Fatty Acids

**DOI:** 10.1038/srep42706

**Published:** 2017-02-15

**Authors:** Esben T. Vestergaard, Niels Jessen, Niels Møller, Jens Otto Lunde Jørgensen

**Affiliations:** 1 Medical Research Laboratory, Aarhus University, Nørrebrogade 44 building 3B, 8000 Aarhus C, Denmark; 2 Department of Endocrinology and Internal Medicine, Aarhus University Hospital, Nørrebrogade 44 building 2A, 8000 Aarhus C, Denmark; 3 Department of Pediatrics, Randers Regional Hospital, Randers 8930 Denmark; 4 Research Laboratory for Biochemical Pathology, Department of Clinical Medicine, Aarhus University Hospital, Nørrebrogade 44 building 3A, 8000 Aarhus C, Denmark

## Abstract

Ghrelin produced in the gut stimulates GH and ACTH secretion from the pituitary and also stimulates appetite and gastric emptying. We have shown that ghrelin also induces insulin resistance via GH-independent mechanisms, but it is unknown if this effect depends on ambient fatty acid (FFA) levels. We investigated the impact of ghrelin and pharmacological antilipolysis (acipimox) on insulin sensitivity and substrate metabolism in 8 adult hypopituitary patients on stable replacement with GH and hydrocortisone using a 2 × 2 factorial design: Ghrelin infusion, saline infusion, ghrelin plus short-term acipimox, and acipimox alone. Peripheral and hepatic insulin sensitivity was determined with a hyperinsulinemic euglycemic clamp in combination with a glucose tracer infusion. Insulin signaling was assayed in muscle biopsies. Peripheral insulin sensitivity was reduced by ghrelin independently of ambient FFA concentrations and was increased by acipimox independently of ghrelin. Hepatic insulin sensitivity was increased by acipimox. Insulin signaling pathways in skeletal muscle were not consistently regulated by ghrelin. Our data demonstrate that ghrelin induces peripheral insulin resistance independently of GH, cortisol, and FFA. The molecular mechanisms remain elusive, but we speculate that ghrelin is a hitherto unrecognized direct regulator of substrate metabolism. We also suggest that acipimox *per se* improves hepatic insulin sensitivity.

Ghrelin is the endogenous ligand for the growth hormone (GH) secretagogue receptor (GHS-R)^1^ and potently stimulates the release of GH and - to a lesser degree - ACTH from the anterior pituitary gland^2^.

It is well documented that GH is lipolytic and induces insulin resistance in skeletal muscle and liver^3^. It is therefore not unexpected that ghrelin administration in healthy subjects is associated with hyperglycemia and increased lipolysis^4^,^5^. The presence of GHS-R in skeletal muscle, adipose tissue, and liver^6^,^7^ suggests that ghrelin also exerts direct tissue effects. In support of this, we have previously demonstrated that ghrelin acutely induces insulin resistance in skeletal muscle independently of GH and cortisol^8^. We also observed that free fatty acid (FFA) concentrations and lipolysis increased in response to ghrelin administration^8^, which is noteworthy since FFAs are known to induce insulin resistance also in the context of GH exposure^9^.

The aim of the present study was to further investigate the direct peripheral effects of ghrelin on insulin sensitivity and substrate metabolism in the presence and absence of concomitant suppression of lipolysis by means of acipimox administration, which suppresses lipolysis and lowers serum FFA levels via inhibition of the hormone sensitive lipase (HSL)^10^. We studied hypopituitary patients on stable replacement therapy with GH and hydrocortisone in order to control for the effects of ghrelin on GH and ACTH release.

## Research Design and Methods

The study was conducted in accordance with the Helsinki Declaration and all subjects gave their oral and written informed consent to participate. The local Ethics Committee and the Danish Medicines Agency approved the study protocol and the protocol was registered at Clinicaltrials.gov NCT01209416 before the onset of enrolment.

### Preparation of synthetic ghrelin

Synthetic human acyl ghrelin (GMP-grade human acyl ghrelin; Bachem, Weil am Rhein, Germany) was dissolved in isotonic saline immediately before infusion. The infusion solution was formulated by the hospital pharmaceutical services and complied with GDP and GCP guidelines.

### Subjects

Eight hypopituitary men on stable replacement therapy with daily sc GH injections in the evening and oral hydrocortisone for >6 months participated in the study (Table 1). GH deficiency was documented by GH stimulation tests (mean ± SE peak GH levels of 0.57 ± 0.21 (range: 0 to 1.61) μg/l). HbA1c at screening was 5.5 ± 0.1% (37 ± 1 mmol/mol). None of the patients had diabetes or any other concomitant chronic disease. The participants were 53 ± 5 years of age and had a BMI of 30.3 ± 4.6 kg/m^2^.

### Study protocol

All participants were examined on four occasions in a 2 × 2 factorial design separated by a minimum of two weeks. The studies were performed in a quiet, thermoneutral indoor environment. The subjects fasted during the trials, but were allowed oral water intake. The patients emptied their urinary bladder before starting the metabolic study day. All patients continued replacement therapy with GH and hydrocortisone during the study; GH was administered subcutaneously at 2200 hr before the metabolic study day and hydrocortisone was administered at 0800 hr on the metabolic study day using the individual subjects normal replacement doses.

In a double-blind and placebo-controlled crossover study each subject underwent four randomized interventions: Ghrelin infusion (1 pmol/min/kg i.v.) and placebo capsules [Ghr], saline infusion and placebo capsules [Control], Ghrelin infusion and acipimox capsules [Ghr + Aci], and saline infusion and acipimox capsules [Aci]. The ghrelin dose of 1 pmol/min/kg was based on our experience from a previous experiment, where that dose increased FFA levels^11^.

**Figure 1:**
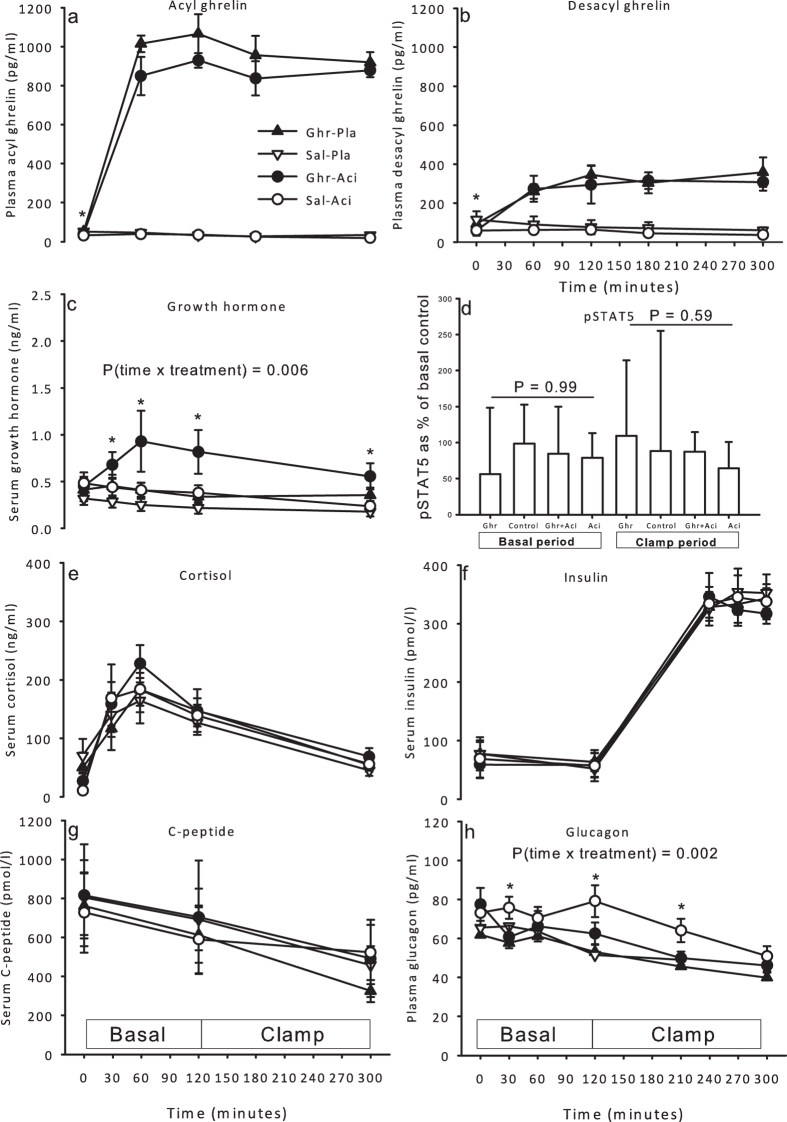
Hormones and phosphorylated STAT5 during ghrelin, saline, ghrelin and acipimox, and acipimox. (**a**) Plasma levels of acyl ghrelin increased in response to ghrelin infusion. (**b**) Plasma levels of desacyl ghrelin at baseline were lower during acipimox treatment. Desacyl ghrelin concentrations increased in response to ghrelin infusion. (**c**) Serum levels of GH increased in response to ghrelin and acipimox treatment. (**d**) Relative levels of pSTAT5 content in skeletal muscle tissue in the basal and in the clamp period. pSTAT5 was similar during all conditions. (**e**) Serum levels of cortisol. (**f**) Serum levels of insulin. (**g**) Serum levels of C-peptide. (**h**) Plasma levels of glucagon increased initially during acipimox treatment and were normalized at the end of the clamp period. Printed P values refer to one-way ANOVA analyses or two-way analyses as indicated. *P < 0.05 at a given time point. All data are presented as mean ± SE.

**Figure 2:**
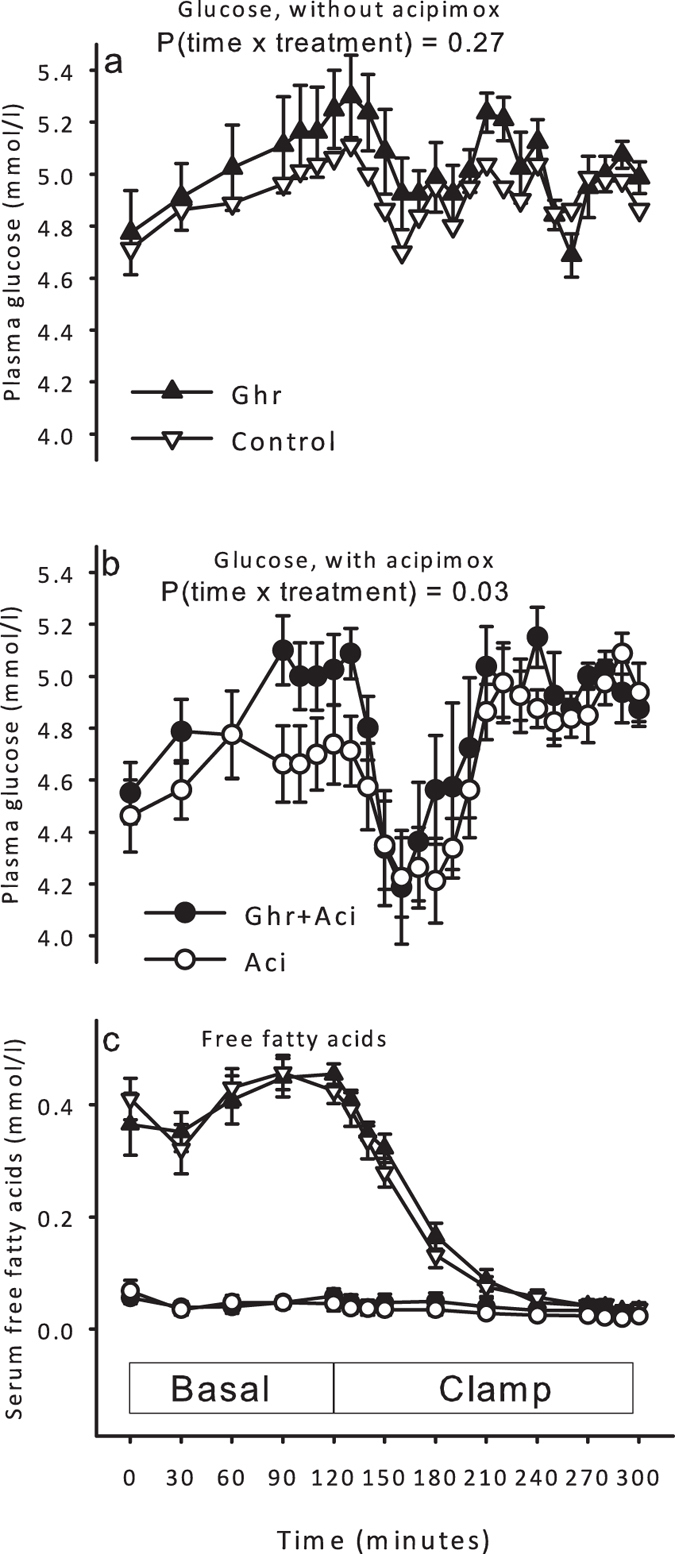
Metabolites during ghrelin, saline, ghrelin and acipimox, and acipimox. (**a**) Plasma glucose during ghrelin and saline infusion. Ghrelin did not impact on plasma glucose. (**b**) Plasma glucose increased during combined ghrelin and acipimox treatment as compared to acipimox-alone. (**c**) Serum free fatty acids. All data are presented as mean ± SE.

In protocol arms Ghr + Aci and Aci, the patients received four doses of acipimox 250 mg, p.o., with two doses administered at 2000 and 2300 hr. the evening before and two doses administered at 0600 and 1000 hr. on the day of the metabolic study. In protocol arms Ghr and Control, the patients received placebo capsules at the same time points. All metabolic studies were performed between 0800 and 1300 hr. (0–300 min) after an overnight fast. One i.v. cannula was inserted into an antecubital region for infusion, and one i.v. cannula was positioned in a dorsal hand vein for blood sampling. The hand was placed in a heat pad in order to arterialize venous blood samples. At t = 0, acyl ghrelin or placebo [isotonic saline (‘Sal’)] infusions as well as a primed (12 μCi) continuous (12 μCi/h) infusion of [3-^3^H] glucose were commenced. The subjects were studied in the basal postabsorptive state (referred to as ‘basal’) for 120 min followed by a hyperinsulinemic/euglycemic clamp (referred to as ‘clamp’) for 180 min, during which they received a constant infusion of insulin (0.6 mU/kg/min; Actrapid, Novo Nordisk, Gentofte, Denmark). Serum insulin was measured at t = 240, 270 and 300 min to document that steady state conditions were achieved. During the insulin infusion, plasma glucose was clamped at ≈5.0 mmol/l by adjusting the rate of infusion of 20% glucose according to plasma glucose measurements carried out every 10 min. Insulin sensitivity was estimated by the level of glucose infusion rate (GIR) during the terminal 30 min of the hyperinsulinemic, euglycemic clamp. Additional blood samples were drawn at the time points as indicated by Figs 1 and 2 and analyzed for acyl and desacyl ghrelin, GH, cortisol, insulin, C-peptide, glucagon, and FFA. Glucose metabolism and indirect calorimetry were assessed during the terminal 30 minutes of both the basal and the clamp period. Skeletal muscle biopsies were obtained t = 30 and 150 min from the lateral vastus muscle with a Bergström biopsy needle under local anesthesia with lidocaine (Xylocain 10 mg/ml; AstraZeneca, Albertslund, Denmark). A total amount of approximately 200 mg muscle was aspirated. Subcutaneous periumbilical adipose tissue biopsies were taken by liposuction technique at t = 30 and 150 min after applying lidocaine as local anesthesia. Biopsies were immediately cleaned for blood, snap-frozen in liquid nitrogen, and stored at −80 °C until analyzed.

The patients voided at t = 120 and 300 min and the urine was measured by volume and a sample was stored at −20 °C for later analysis.

### Biochemical analyses

Plasma glucose was analyzed bedside using the glucose oxidase method (YSI 2300 STAT Plus; YSI Life Sciences, Yellow Springs, OH). Serum and plasma samples were frozen and stored at −20 °C or at −80 °C (ghrelin and glucagon). Serum FFAs were analyzed by a commercial kit (Wako Chemicals, Neuss, Germany). Samples for plasma ghrelin measurements were drawn in 2 ml acidified EDTA prepared vacutainers with 20 μl 200 mg/ml AEBSF (Sigma-Aldrich Denmark A/S, Copenhagen, Denmark) and centrifuged immediately at 2,500 *g* for 10 minutes at 4 °C. AEBSF is a serine protease inhibitor, which is added to the collecting blood tubes to inhibit/reduce breakdown of acyl ghrelin. Plasma was then transferred to 1.8 ml tubes and stored at −80 °C until analysis. Plasma acyl and desacyl ghrelin were determined using ELISAs (Bertin Pharma, Montigny-le-Bretonneux, France; A05106 and A05119, respectively) using a modified protocol according to Delhanty *et al*.^12^ Serum GH was analyzed using chemiluminescence technology (IDS-iSYS Multi-Discipline Automated Analyzer, Immunodiagnostic Systems Nordic a/s Herlev, Denmark). Serum insulin was analyzed using time-resolved fluoroimmunoassay assay (AutoDELFIA Insulin kit, catalog no. B080–101, PerkinElmer, Turku, Finland). Serum cortisol was measured using a DRG ELISA kit (DRG Instruments GmbH, Marburg, Germany). The specific activity of [3-^3^H] glucose was determined as previously described^13^. Rates of glucose appearance (Ra) and disappearance (Rd) were calculated using Steele’s non-steady state equation using a pool fraction of 0.65. Endogenous glucose production (EGP) during the clamp, which is a measure of hepatic insulin sensitivity, was calculated by subtracting the glucose infusion rate from glucose Ra during the terminal 30 min of the clamp. Energy expenditure (EE) and respiratory exchange rate (RER) were calculated by indirect calorimetry using a computerized open circuit system (Deltatrac; Datex Instruments, Helsinki, Finland). Oxidation rates of glucose (GOX) and lipids were calculated from EE and RER after correction for protein oxidation, which was estimated from the urinary excretion of urea. Nonoxidative glucose disposal (NOGD) was calculated as whole body glucose disposal *R*_*d*_ minus the rate of GOX^14^.

Freeze dried muscle biopsies were homogenized at 4 °C in a buffer (pH 7.4) containing 50 mM HEPES, 137 mM NaCl, 10 mM Na_4_P_2_O_7_, 20 mM NaF, 5 mM EDTA, 1 mM MgCl_2_, 1 mM CaCl_2_, 2 mM sodium orthovanadate (NaOV), 5 mM nicotinamide (NAM), 10 μM trichostatin A (TSA), HALT Protease Inhibitor Cocktail, Nonidet P-40 (NP-40) 1%, and 10% glycerol. Samples were centrifuged at 14,000 *g* for 20 minutes.

Western blot analyses were used to measure phosphorylated and total levels of intracellular insulin and GH signaling proteins. The primary antibodies used were from Cell Signaling Technology, Danvers, MA (Akt, pAkt Ser473 and Thr308, pAS160 Thr642, STAT5, pSTAT5 Tyr694, Glycogen Synthase (GS), and pGS Ser641) and Merck Millipore, Darmstadt, Germany (AS160 and GLUT4). Control for equal loading was performed using the stain-free technology^15^. Proteins were visualized and quantified using Image Lab 5.0, Bio-Rad laboratories (BioRad, CA). Quantifications are expressed as the ratio between phosphorylated protein and the total protein measurement on the same membranes. Differences between interventions are expressed as the ratio change from the measurement made in the basal period of the placebo day for each subject.

Skeletal muscle glycogen content: Muscle samples were hydrolyzed in 2 M HCl at 100 °C for 2 h, followed by neutralization with 2 M NaOH^16^, and glucose content was measured by the hexokinase enzymatic method using a glucose hexokinase reagent (Eagle Diagnostics, Desoto, TX)^17^.

### Statistical analysis

Results are expressed as mean ± standard error of the mean (mean ± SE) or median and 25–75 percentile. The statistical analyses were performed by using SigmaPlot 11.0 (©Systat Software, CA). A two way-ANOVA (time × treatment) for repeated measurements with Student-Newman-Keuls post-hoc analysis was used to test for significant differences in time series and the GIR. Concentrations at single time points were analyzed by a Student’s two-tailed paired *t* test or a one-way ANOVA depending on the number of variables or a Signed Rank Test, if data was not normally distributed. A *P* value <0.05 was considered significant.

## Results

### Hormones

Circulating hormone concentrations are shown in Fig. 1. Baseline plasma levels of ghrelin (pg/ml) were lower during acipimox: 49.6 ± 9.4 [Ghr], 51.9 ± 15.1 [Control], 39.4 ± 7.8 [Ghr + Aci], and 31.6 ± 8.2 [Aci] *P* = 0.045, and plasma ghrelin increased approximately 20 fold during ghrelin infusion (Fig. 1a). Baseline plasma levels of unacylated ghrelin (UAG, pg/ml) were also lower during acipimox: 93.4 ± 34.3 [Ghr], 115.4 ± 45.5 [Control], 58.8 ± 25.4 [Ghr + Aci], and 59.5 ± 20.6 [Aci] *P* = 0.02, and increased approximately 6 fold during ghrelin infusion (Fig. 1b). Serum GH levels increased slightly but significantly during Ghr + Aci (*P* = 0.006), but were not increased during Ghr alone (Fig. 1c). The small increase in GH levels during Ghr + Aci did not translate into detectable GH-signal transduction as measured by phosphorylation of STAT5 at Tyr694 in skeletal muscle tissue (Fig. 1d and Supplemental Figure S1). Serum cortisol levels were similar during all 4 conditions (*P* = 0.48, Fig. 1e). Serum insulin levels increased during the clamp, but were not affected by treatment (Fig. 1f), and serum C-peptide levels decreased during insulin infusion, but independently of treatment (Fig. 1g). Plasma glucagon levels interacted significantly with time and treatment (*P* = 0.002) and were increased by acipimox (Fig. 1h).

### Metabolites

Plasma glucose levels were similar at baseline and in the basal period without acipimox exposure: [Ghr] vs. [Control] *P* = 0.27 (Fig. 2a), whereas concomitant acipimox exposure caused basal plasma glucose concentrations to increase in response to ghrelin infusion (*P* = 0.03, Fig. 2b).

Acipimox suppressed serum FFA levels (mmol/l): 0.37 ± 0.06 [Ghr], 0.41 ± 0.04 [Control], 0.06 ± 0.01 [Ghr + Aci], and 0.07 ± 0.02 [Aci] (*P* < 0.01), but ghrelin infusion did not impact on FFA levels ([Ghr] vs. [Control] *P* = 0.46, Fig. 2c).

### Insulin sensitivity and glucose turnover

**Figure 3:**
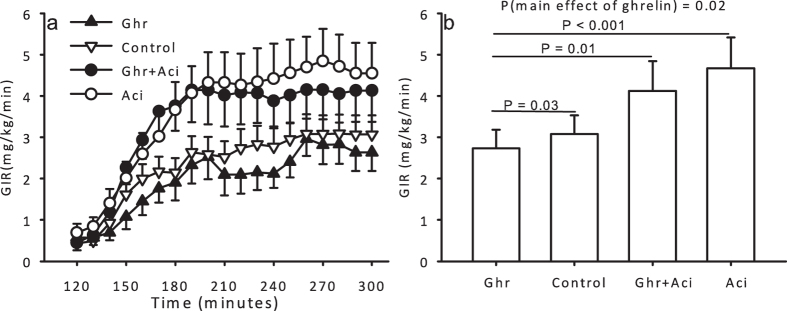
(**a**) Glucose infusion rates during ghrelin, saline, ghrelin and acipimox, and acipimox. (**b**) GIR the four treatment conditions. Printed P values refer to paired *t* tests or one-way ANOVA where indicated. All data are presented as mean ± SE.

Ghrelin infusion induced peripheral insulin resistance, as assessed by the *GIR*, independently of acipimox (*P* = 0.02), whereas acipimox improved peripheral insulin sensitivity independently of ghrelin (*P* = 0.005, Fig. 3a and b). Post hoc pairwise comparison revealed an insulin antagonistic effect of ghrelin as compared to saline ([Ghr] vs. [Control], *P* = 0.03).

**Figure 4:**
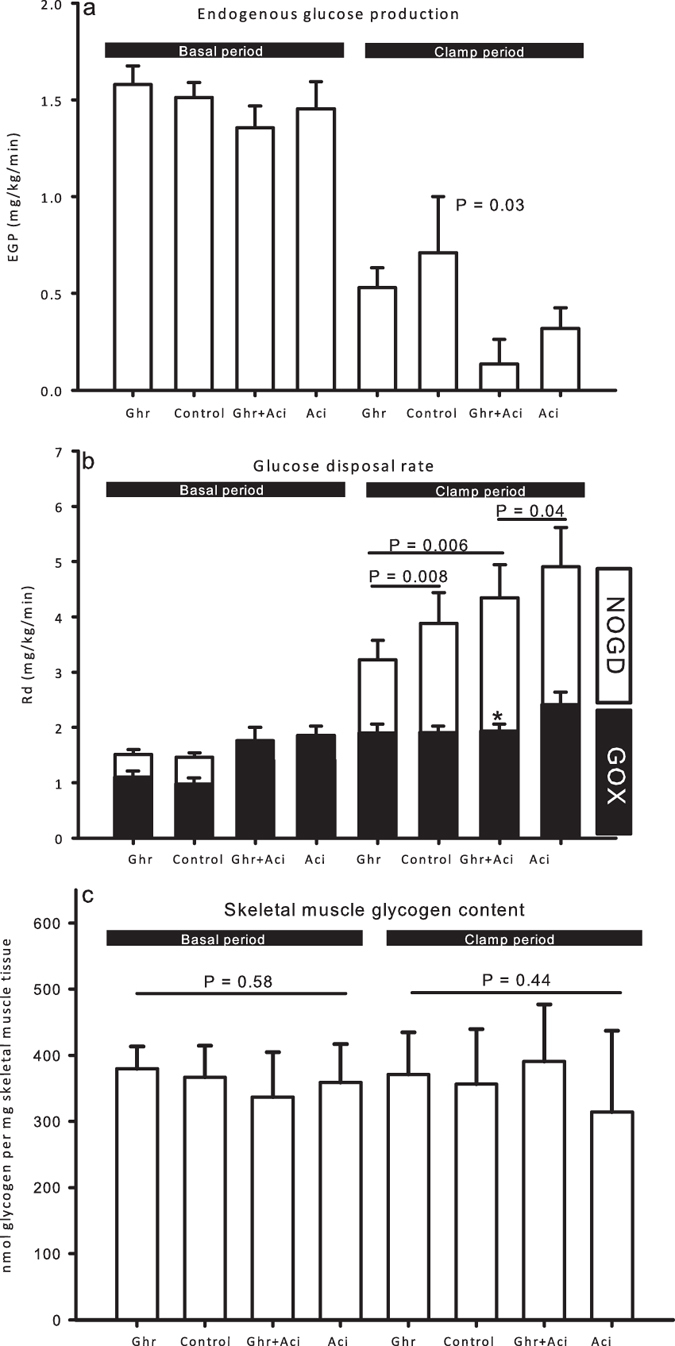
Glucose metabolism during ghrelin, saline, ghrelin and acipimox, and acipimox-alone in the basal and in the clamp periods. (**a**) EGP was equal in the basal period during all four conditions. During the clamp EGP was suppressed by acipimox. Printed P value refers to two-way ANOVA treatment effect of acipimox. (**b**) Glucose utilization during the terminal 30 min of basal and clamp periods. Glucose metabolism was similar in the basal period. During the clamp, ghrelin reduced glucose disposal both with and without acipimox treatment. Acipimox reversed the suppressive effect of ghrelin on glucose disposal during the clamp. Printed P values refer to paired *t* tests. (**c**) Skeletal muscle glycogen content. Printed P values refer to one-way ANOVA tests. All data are presented as mean ± SE.

Ghrelin and acipimox did not affect EGP in the basal period (Fig. 4a). Hepatic insulin sensitivity, as determined by EGP during the clamp, was not affected by ghrelin alone (*P* = 0.25), whereas acipimox significantly decreased EGP independently of ghrelin (acipimox main effect *P* = 0.03, Fig. 4a).

Ghrelin did not significantly impact glucose turnover during the basal period (Fig. 4b). In the clamp period, ghrelin reduced glucose disposal (mg/kg/min) both in the absence of acipimox (3.22 ± 0.35 [Ghr] vs. 3.88 ± 0.56 [Control]*, P* = 0.008) and in the presence of acipimox (4.34 ± 0.60 [Ghr + Aci] vs. 4.91 ± 0.71 [Aci], *P* = 0.04). However, acipimox significantly counteracted the suppressive effect of ghrelin on glucose disposal (Fig. 4b). Oxidative glucose disposal (GOX) was reduced during ghrelin plus acipimox as compared to acipimox alone (Fig. 4b). This was not associated with detectable changes in GLUT4 protein levels in skeletal muscle biopsies (data not shown). Post hoc analyses showed that the effects of ghrelin on glucose turnover were most pronounced with regards to suppression of non-oxidative glucose disposal (NOGD) (Fig. 4b), but this did not result in differences in skeletal muscle glycogen content (nmol glycogen/mg muscle) (399.9 ± 26.1 [Ghr] vs. 336.2 ± 51.1 [Control] vs. 394.9 ± 27.5 [Ghr + Aci] vs. 305.2 ± 52.4 [Aci], NS, Fig. 4c). In agreement with unaltered glycogen content, phosphorylation of glycogen synthase at the activity-regulating phosphorylation site on Ser641 was similar between groups (data not shown).

**Figure 5:**
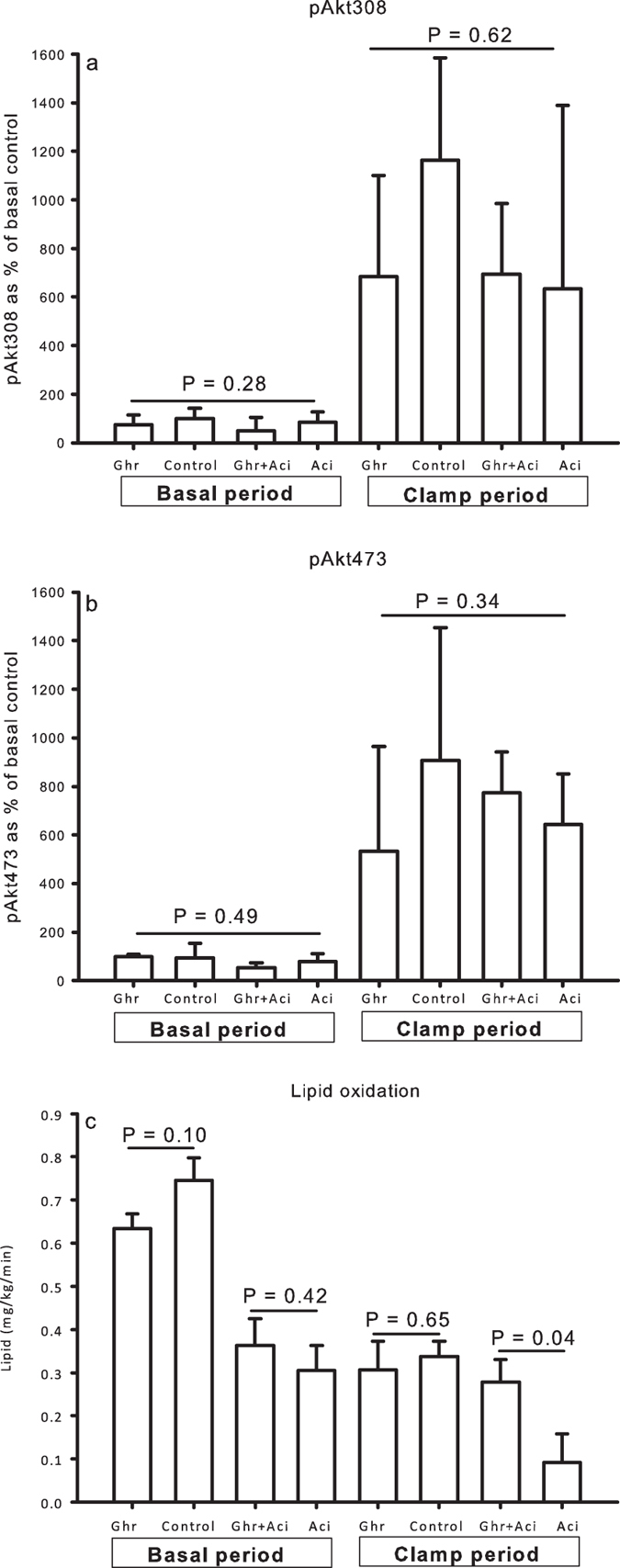
Phosphorylated enzymes in the insulin signaling cascade and lipid oxidation. (**a** and **b**) Ghrelin did not impact on relative levels of pAkt308 and -473 content in skeletal muscle tissue in the basal and in the clamp period. (**c**) Ghrelin antagonized the suppressive effect of acipimox on lipid oxidation during the clamp. Printed P values refer to one-way ANOVA tests (**a** and **b**) or paired *t* tests (C). All data are presented as mean ± SE.

Insulin infusion induced a significant (*P* < 0.01) 8-fold increase in pAkt expression at Thr308 and Ser473 (Fig. 5a and b and Supplemental Figure S1). This was associated with an increased downstream signaling to AS160 in terms of a 4-fold increase in phosphorylation at Thr642 during insulin stimulation (*P* < 0.01) (data not shown). However, this effect of insulin was not modified by either ghrelin or acipimox.

### Resting energy expenditure and lipid oxidation

REE (kcal/24-h) was comparable in the basal period (1731 ± 138 [Ghr] vs. 1794 ± 111 [Control] vs. 1713 ± 120 [Ghr + Aci] vs. 1720 ± 131 [Aci], *P* = 0.13) and in the clamp period (1784 ± 124 [Ghr] vs. 1825 ± 106 [Control] vs. 1838 ± 122 [Ghr + Aci] vs. 1803 ± 91 [Aci], *P* = 0.64). Ghrelin did not significantly influence RER in the basal period ([Ghr] vs. [Control]: *P* = 0.40; [Ghr + Aci] vs. [Aci]: *P* = 0.40), whereas the RER increased during acipimox in the basal period (0.83 ± 0.01 [Ghr] vs. 0.81 ± 0.01 [Control] vs. 0.88 ± 0.02 [Ghr + Aci] vs. 0.90 ± 0.01 [Aci], *P* < 0.001). Ghrelin had no influence on the RER in the clamp period in the absence of acipimox ([Ghr] vs. [Control]: *P* = 0.77). The RER also increased during acipimox exposure in the clamp period (0.90 ± 0.01 [Ghr] vs. 0.89 ± 0.01 [Control] vs. 0.90 ± 0.01 [Ghr + Aci] vs. 0.94 ± 0.02 [Aci], *P* = 0.03); this effect was abrogated by ghrelin ([Ghr + Aci] vs. [Aci]: *P* = 0.03).

Ghrelin had no significant effects on lipid oxidation rates (mg/kg/min) in the absence of acipimox (0.31 ± 0.07 [Ghr] vs. 0.34 ± 0.07 [Control], *P* = 0.65), but ghrelin significantly antagonized the suppressive effect of acipimox on lipid oxidation (0.28 ± 0.05 [Ghr + Aci] vs. 0.09 ± 0.07 [Aci], *P* = 0.04, Fig. 5c).

## Discussion

The present study was designed to investigate whether ghrelin-induced insulin resistance depends on lipolysis and ambient FFA levels after correction for ghrelin-induced stimulation of GH and cortisol release. Our data demonstrate that ghrelin *per se* induces peripheral insulin resistance independently of ambient FFA levels.

We have previously reported that ghrelin infusion increases serum FFA levels in hypopituitary patients which could imply a direct lipolytic effect^8^. This was not reproduced in the present study, where serum FFA levels during ghrelin infusion vs. saline were similar. It is possible that this discrepancy reflects a dose-dependent lipolytic effect of ghrelin, inasmuch as the dose employed in the present study was 5-fold lower and thus more physiological as compared to our previous studies^5^,^8^,^18^. It remains to be experimentally tested whether ghrelin stimulates lipolysis or FFA turnover in a dose-dependent manner, but regardless of that, our data reveals a direct FFA-independent effect of ghrelin on peripheral insulin sensitivity.

Acipimox is an antilipolytic nicotinic acid analogue, which binds to a G-protein coupled receptor – “*protein upregulated in macrophages by interferon-γ”* (PUMA-G/HM74) – in adipose tissue and thereby inhibits the hormone sensitive lipase (HSL)^19^,^20^ resulting in acute suppression of systemic FFA levels and improved peripheral insulin sensitivity^21^. The present study documents these effects and confirms that acipimox also improves hepatic insulin sensitivity^22^. The latter effect of acipimox is unmasked by our model with stabilized GH levels, inasmuch as acipimox is known to increase GH secretion^23^,^24^, which in turn induces hepatic (and peripheral) insulin resistance^25^,^26^. We therefore suggest that our model is well suited to study the direct effect of acipimox and also propose an explanation as to why hepatic and peripheral insulin sensitivity frequently fails to improve following acipimox administration to subjects with an intact anterior pituitary function^23^,^27^. In line with previous studies we recorded a stimulatory effect of acipimox on plasma glucagon concentrations^9^,^23^, which may represent a compensatory mechanism.

Skeletal muscle uptake accounts for approximately 80% of infused glucose during a hyperinsulinemic euglycemic clamp in humans^28^, but it has proven difficult to demonstrate a link between the diabetogenic effects of ghrelin to the insulin signaling pathway in human skeletal muscle biopsies^8^,^11^. Likewise, no evidence of impaired insulin signaling at the level of protein expression or phosphorylation could be recorded in the present study. It is possible that more frequent biopsies in combination with activity assays might provide additional sensitivity to our human *in vivo* model, but it is also possible that *in vitro* studies or transgenic mice models may be necessary to unveil the molecular mechanism.

Ghrelin infusion increased both acyl ghrelin and desacyl ghrelin levels to supraphysiologic concentrations in line with earlier reports^11^,^29^,^30^, which supports that acyl ghrelin is metabolized to desacyl ghrelin *in vivo*. Surprisingly, acipimox reduced both ghrelin and desacyl ghrelin concentrations at baseline, which is interesting since desacyl ghrelin exposure has been shown to improve insulin sensitivity^31^,^32^,^33^.

Both ghrelin and acipimox are recognized as potent GH secretagogues^24^. Our participants all had documented GH deficiency as a consequence of pituitary disease, yet we did record a minimal increase in serum GH levels when ghrelin and acipimox were co-administered. This observation indicates synergy between the GH secreting effects of ghrelin and acipimox and is in line with effects reported in healthy elderly men treated with acipimox and the GH-secretagogue GHRP-2^34^. This response could theoretically have impacted on glucose and lipid metabolism, but it was not accompanied by detectable activation of GH signaling in skeletal muscle. Taken together, we find it unlikely that it could account for the metabolic effects of ghrelin observed in our study.

Baseline plasma glucose concentrations were not increased by ghrelin alone, which is in line with previous reports^29^ but plasma glucose levels during administration of ghrelin plus acipimox were elevated as compared to the levels recorded after acipimox alone, which appeared to increase plasma glucagon levels. This lack of effect of ghrelin on glucose concentrations contrasts with our earlier data^5^,^8^,^18^, but, again, in the present study we used a more physiologic ghrelin infusion rate and the present data are in line with other clinical trials using a similar ghrelin infusion rate^11^. Plasma insulin and ghrelin appear to correlate inversely. Physiological ghrelin concentrations inhibit insulin secretion^35^,^36^ and vice versa. It is speculated that lower ghrelin levels in obesity^38^,^39^ and type 2 diabetes^40^ may represent a counter-regulatory mechanism against hyperglycemia.

Some limitations apply to our study. The number of participants was relatively small, the age and BMI ranges were relatively large, and the study included male patients only. However, the crossover design minimizes inter-individual differences and ensures that the observed effects of ghrelin and acipimox are not attributable to differences in the study population.

In conclusion, our data show that ghrelin induces acute peripheral insulin resistance via mechanisms that are independent of GH, cortisol, and ambient serum FFA levels but does not impact on hepatic insulin sensitivity.

## Additional Information

**How to cite this article**: Vestergaard, E. T. *et al*. Acyl Ghrelin Induces Insulin Resistance Independently of GH, Cortisol, and Free Fatty Acids. *Sci. Rep.* **7**, 42706; doi: 10.1038/srep42706 (2017).

**Publisher's note:** Springer Nature remains neutral with regard to jurisdictional claims in published maps and institutional affiliations.

## Supplementary Material

Supplemental Figure S1

## Figures and Tables

**Table 1:** Characterization of the subjects.

Patient	Age yr	BMI kg/m^2^	Diagnosis	Diagnostic test	GH peak μg/l	GH dose mg daily	HbA1c		Insufficient pituitary axes
							mmol/mol	%	
1	68	32.8	Pituitary apoplexy	Insulin tolerance test	0.08	0.15	34	5.3	GH, T, C, Gn
2	67	26.4	Pituitary apoplexy	Insulin tolerance test	0.99	0.4	41	5.9	GH, T, C, Gn
3	26	26.0	Congenital hypopituitaism	GHRH plus arginine stimulation test	1.61	0.3	36	5.4	GH, T, C
4	48	31.7	Pituitary apoplexy	Insulin tolerance test	0	0.5	39	5.7	GH, T, C, Gn
5	67	27.0	Pituitary adenoma, surgical treatment and radiotherapy	Insulin tolerance test	0.41	0.2	41	5.9	GH, T, C, Gn
6	52	30.4	Clinically nonfunctioning pituitary adenoma	Arginine stimulation test	1.12	0.3	40	5.8	GH, T, C, Gn
7	50	25.8	Pituitary cyst, surgical treatment	Insulin tolerance test	0.20	0.3	33	5.2	GH, T, C, Gn
8	42	42.0	Traumatic brain injury	Arginine stimulation test	0.13	0.3	30	4.9	GH, T, C, Gn

BMI, body mass index; IGF-I levels at baseline during GH substition. GH, growth hormone; T, thyrotropin; C, corticotropin; Gn, gonadotropin.
