# Supplementary material for: Acyl Ghrelin Induces Insulin Resistance Independently of GH, Cortisol, and Free Fatty Acids
Source: Sci Rep. 2017 Feb 15;7:42706. doi: 10.1038/srep42706 (PMC5309923; doi:10.1038/srep42706)
Supplement: Supplemental Figure S1 [file srep42706-s1.pdf]

Acyl Ghrelin Induces Insulin Resistance Independently of GH, Cortisol, and Free Fatty Acids

Esben T Vestergaard MD, PhD,[1,2,3] Niels Jessen, MD, PhD,[1,2,4] Niels Møller, Professor, MD, DMSc,[1,2] and Jens Otto Lunde Jørgensen, Professor, MD, DMSc[1,2]

Affiliation

[1]Medical Research Laboratory, Aarhus University, Nørrebrogade 44 building 3B, 8000 Aarhus C, Denmark. [2]Department of Endocrinology and Internal Medicine, Aarhus University Hospital, Nørrebrogade 44 building 2A, 8000 Aarhus C, Denmark. [3]Department of Pediatrics, Randers Regional Hospital, 8930 Randers. [4]Research Laboratory for Biochemical Pathology, Department of Clinical Medicine, Aarhus University Hospital, Nørrebrogade 44 building 3A, 8000 Aarhus C, Denmark.

Clinical trials registration number: Clinicaltrials.gov NCT01209416 date of registration September 24, 2010

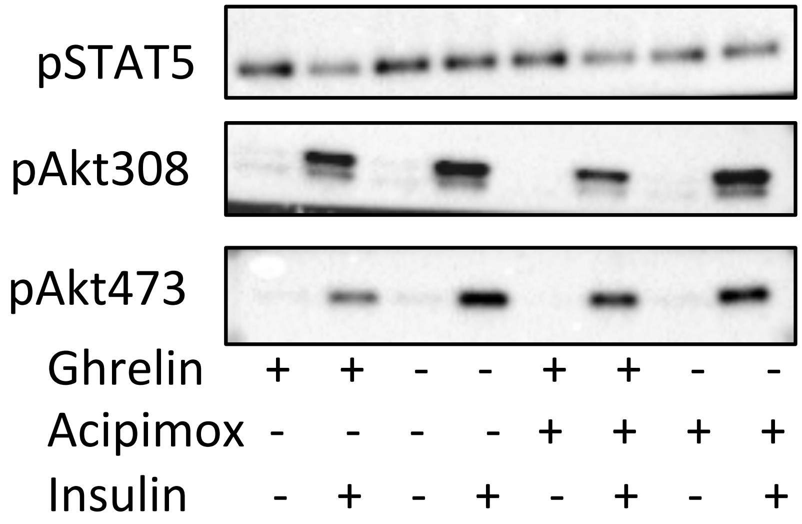

Supplemental Figure S1. Representative Western blots of pSTAT5, pAkt308, and pAkt473 in skeletal muscle in hypopituitary patients during ghrelin and saline infusion and during acipimox and placebo treatment both with and without insulin exposure.
